# Effects of Unilateral Nephrectomy on Renal Function in Male Spontaneously Diabetic Torii Fatty Rats: A Novel Obese Type 2 Diabetic Model

**DOI:** 10.1155/2014/363126

**Published:** 2014-08-10

**Authors:** Yoshiaki Katsuda, Yusuke Kemmochi, Mimi Maki, Ryuhei Sano, Yasufumi Toriniwa, Yukihito Ishii, Katsuhiro Miyajima, Kochi Kakimoto, Takeshi Ohta

**Affiliations:** ^1^Japan Tobacco Inc., Central Pharmaceutical Research Institute, Biological/Pharmacological Research Laboratories, 1-1 Murasaki-cho, Takatsuki, Osaka 569-1125, Japan; ^2^Japan Tobacco Inc., Central Pharmaceutical Research Institute, Toxicology Research Laboratories, 23 Naganuki, Hadano, Kanagawa 257-0024, Japan

## Abstract

The Spontaneously Diabetic Torii (SDT) fatty rat is a new model for obese type 2 diabetes. The aim of the present study was to investigate the effect of 1/2 nephrectomy (Nx) on renal function and morphology and on blood pressure in SDT fatty rats. Male SDT fatty rats underwent 1/2 Nx or a sham operation (Sham). Subsequently, animals were studied with respect to renal function and histological alterations. Induction of 1/2 Nx in SDT fatty rats led to functional and morphological damage to the remnant kidney and to hypertension, which are considered main characteristics of chronic kidney disease, at a younger age compared with the sham group. In conclusion, the SDT fatty rat is useful in investigations to elucidate the pathogenesis of human diabetic nephropathy and in new drug discovery.

## 1. Introduction

Chronic kidney disease (CKD) is a worldwide public health problem associated with significant morbidity and mortality [1]. Several risk factors contribute to the development and progression of CKD, including hypertension, diabetes, and dyslipidemia [2–4]. In particular the increase in number of patients with obesity-associated type 2 diabetes has resulted in a rapid increase in patients who have end stage renal disease (ESRD) and require dialytic life support [5, 6]. Despite efforts to develop means to prevent or arrest the progression of the disease, long-term prognosis of patients with established nephropathy remains poor [7]. Diabetic nephropathy has been recognized as a primary disease of CKD and the investigation of diabetic nephropathy is essential for the understanding of the pathogenesis of CKD.

Diabetic animal models have a critical role in the elucidation of mechanisms of diabetic complications and the development of novel drugs as treatments. Consequently, the understanding of the pathophysiology of renal lesions in diabetic models is beneficial in the design and development of therapies.

The Spontaneously Diabetic Torii (SDT) fatty rat, established by introducing the* fa* allele in Zucker fatty rats into the SDT rat genome, is a model for obese type 2 diabetes showing overt obesity, hyperglycemia, and hyperlipidemia from a younger age and resulting in early onset of diabetic complications [8–12]. Furthermore, SDT fatty rats showed elevated blood pressure, in addition to the aforementioned metabolic abnormalities [13, 14]. Therefore, the SDT fatty rat is considered to be a useful model for the analysis of diabetes and related complications such as diabetic nephropathy.

In this study, we investigated the effects of unilateral nephrectomy on renal function and morphology in SDT fatty rats.

## 2. Materials and Methods

### 2.1. Animals

This experiment was conducted in compliance with the Guidelines for Animal Experimentation established for our biological/pharmacological laboratories. Male SDT fatty rats from Japan Tobacco colonies were used in this study. Animals were divided into 2 groups: those undergoing 1/2 nephrectomy (1/2 Nx) or sham operation (sham). Animals at 8 weeks of age underwent 1/2 Nx or sham surgery under anesthesia. A small lumbar incision was made, and the left kidney was removed. In sham-operated animals, the left kidney was exposed and gently manipulated but left intact. Animals were housed in suspended bracket cages and given a standard laboratory diet (CRF-1; Oriental Yeast Co., Ltd., Tokyo, Japan) in a room with controlled temperature, humidity, and lighting.

### 2.2. Biological Parameters

Body weight, biochemical parameters, and renal parameters were assessed from 10 to 18 weeks of age, every 2 weeks. Blood samples were collected from the tail vein of nonfasted rats. Serum glucose, triglyceride (TG), and total cholesterol (TC) were measured as a biochemical parameter using commercial kits (F. Hoffmann-La Roche Ltd., Basel, Switzerland) and an automatic analyzer (Hitachi, Ltd., Tokyo, Japan).

Urine volume, urinary albumin, blood urea nitrogen (BUN), and creatinine clearance (Ccr) were evaluated as renal parameters. Urine samples were collected by placing the animals in metabolic cages with water for 6 h. Urinary albumin level was measured with a rat-albumin enzyme immunoassay (EIA) kit (Panapharm Laboratories Co., Ltd., Kumamoto, Japan). Urinary creatinine, serum creatinine, and BUN were measured using commercial kits (Roche Diagnostics, Basel, Switzerland) and an automatic analyzer (Hitachi, Ltd., Tokyo, Japan). Ccr was calculated by dividing the 6 h urinary excretion of creatinine by serum creatinine level and body weight.

Systolic blood pressure (SBP) and heart rate in a conscious nonfasted state were measured at 12 and 16 weeks of age by the indirect tail cuff method using a Softron BP-98A indirect blood pressure meter (Softron Co. Ltd., Tokyo, Japan). Blood pressure and heart rate were measured between 13:00 and 16:00 hours. Five measurements were taken for each rat and subsequently averaged.

### 2.3. Histopathological Examination

Necropsy was performed at 18 weeks of age and kidneys were collected from all animals. Kidneys were weighed and fixed with 4% paraformaldehyde. After resection, the tissues were paraffin-embedded using standard techniques and thin-sectioned (3–5 *μ*m). Sections were stained with hematoxylin and eosin (HE) and periodic acid Schiff (PAS). Eight mice in the 1/2 Nx group and five mice in the sham group were examined histopathologically in a blinded manner.

### 2.4. Statistical Analysis

Results of biological parameters were expressed as means ± standard deviation (SD). Statistical analyses of differences between mean values in the sham group and the 1/2 Nx group were performed using the *F*-test, followed by the Student's* t*-test or Aspin-Welch's* t*-test. Differences were defined as significant when *P* < 0.05.

## 3. Results

### 3.1. Body Weight and Biochemical Parameters

Body weights in the 1/2 Nx group were comparable to those in the sham group from 10 to 18 weeks of age (1/2 Nx group; 479.7 ± 116.2 g at 18 weeks of age, sham group; 521.2 ± 40.3 g at 18 weeks of age). The 1/2 Nx group and sham group had similar levels of plasma glucose from 10 to 18 weeks of age (1/2 Nx group, 751.0 ± 63 mg/dL at 18 weeks of age; sham group, 799.9 ± 76.9 mg/dL at 18 weeks of age). Serum TG levels in the 1/2 Nx group at 18 weeks of age were significantly higher than those in the sham group (1/2 Nx group, 501.9 ± 211.4 mg/dL at 18 weeks of age; sham group, 309.6 ± 67.4 mg/dL at 18 weeks of age). Serum TC levels tended to increase in the 1/2 Nx group (1/2 Nx group, 154.4 ± 51.7 mg/dL at 18 weeks of age; sham group, 120.8 ± 18.7 mg/dL at 18 weeks of age).

### 3.2. Renal Parameters

**Figure 1:**
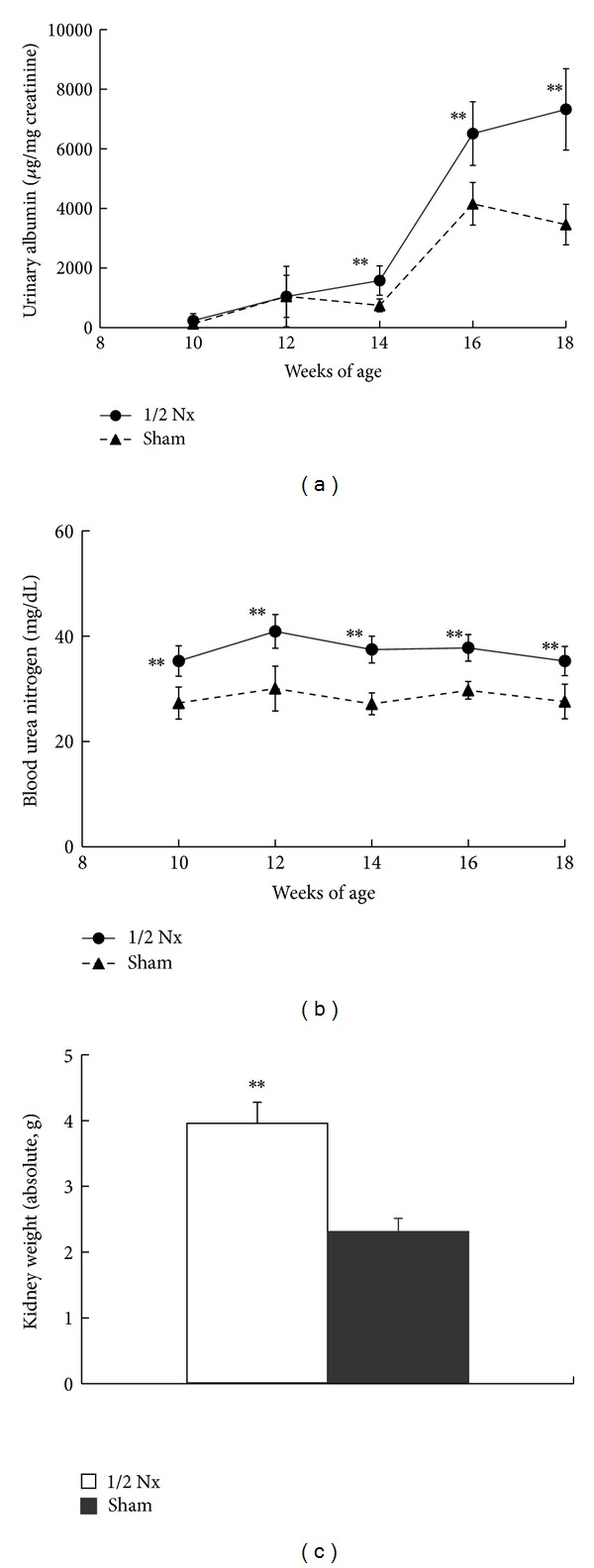
Changes in renal parameters ((a) urinary albumin, (b) blood urea nitrogen, and (c) kidney weight) in the 1/2 Nx group and sham group. Data shown as means ± SD ((a) and (b) *n* = 8–10 and (c) *n* = 8-9). **P < 0.01; significantly different from the sham group.

Urinary albumin in the 1/2 Nx group increased from 14 weeks of age compared with those in the sham group (Figure 1(a)). Serum BUN levels in the 1/2 Nx group were significantly higher than those in the sham group during the experimental period (Figure 1(b)). Kidney weights in the 1/2 Nx group were higher compared with those in the sham group (Figure 1(c)). Ccr tended to decrease in the 1/2 Nx group (1/2 Nx group, 0.23 ± 0.12 mL/h∗g at 18 weeks of age; sham group, 0.31 ± 0.06 mL/h∗g at 18 weeks of age). Urine volumes in the 1/2 Nx group were comparable to those in the sham group from 10 to 18 weeks of age (1/2 Nx group, 13.17 ± 6.58 mL at 18 weeks of age, 0.23 ± 0.12 mL/h∗g; sham group, 15.90 ± 6.11 mL at 18 weeks of age).

### 3.3. Blood Pressure

**Figure 2:**
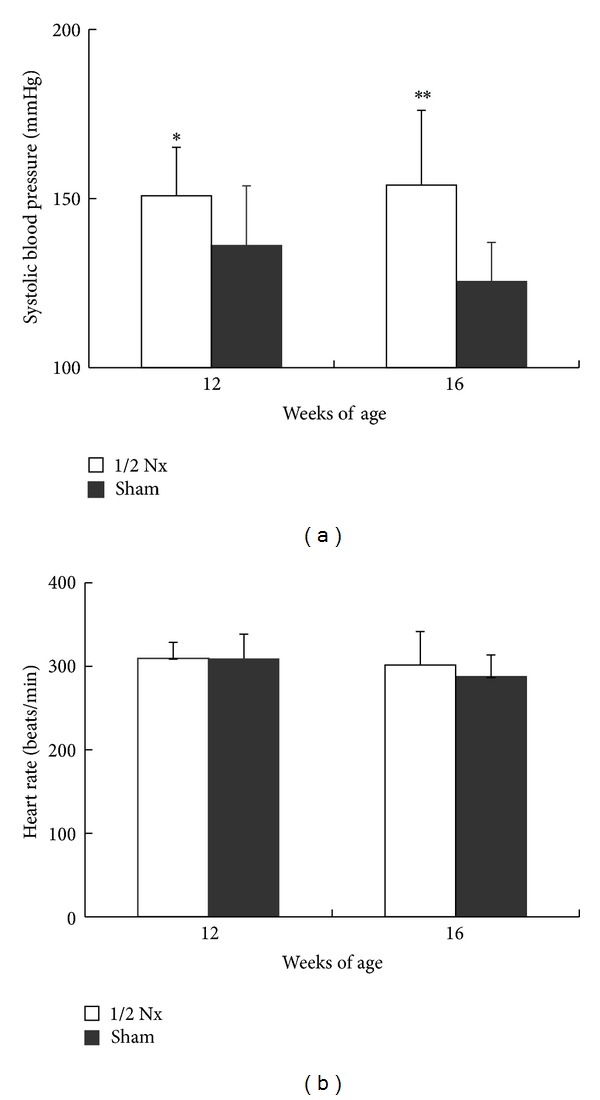
Systolic blood pressure (a) and heart rate (b) at 12 and 16 weeks of age in the 1/2 Nx group and sham group. Data shown as means ± SD (*n* = 8–10). *P < 0.05, **P < 0.01; significantly different from the sham group.

SBP levels at 12 and 16 weeks of age in the 1/2 Nx group were significantly elevated compared with those in the sham group (Figure 2(a)). For heart rate, there were no differences among groups (Figure 2(b)).

### 3.4. Histopathological Examinations of the Kidney

**Figure 3:**
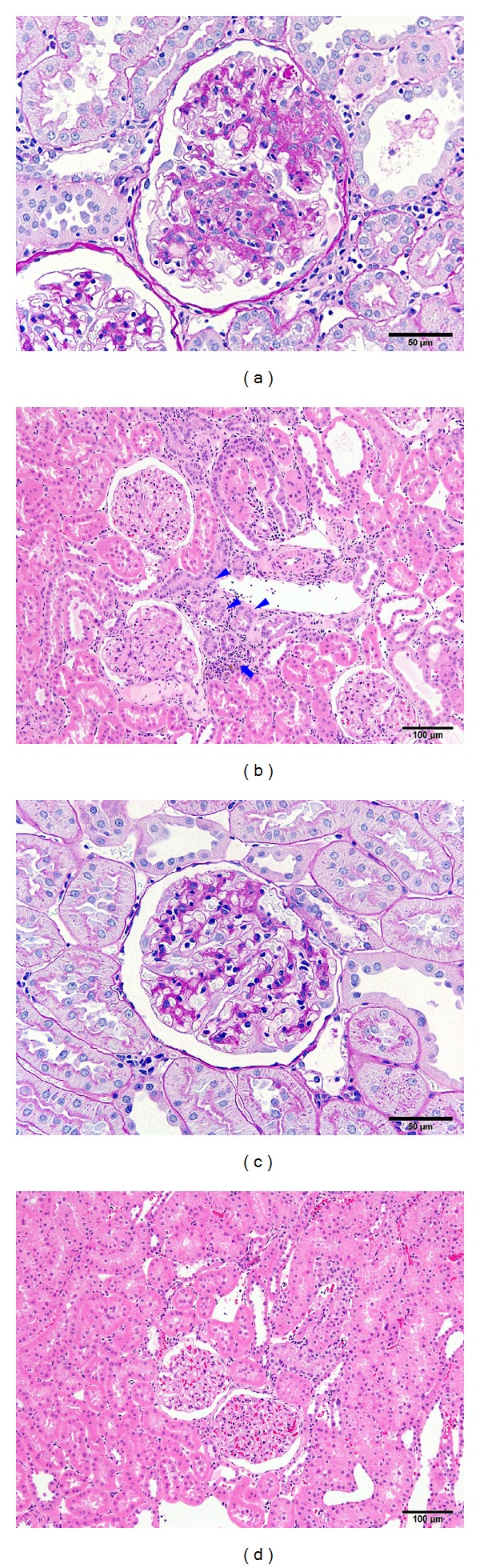
Photomicrograph of kidney tissues from the 1/2 Nx group (a) and (b) and sham group (c) and (d). Marked glomerulosclerosis (a) tubular regeneration (arrowhead in (b)) and inflammatory cell infiltration (arrow in (b)) observed in the 1/2 Nx group. Periodic acid Schiff ((a) and (c)), bar = 50 *μ*m. Hematoxylin and eosin ((b) and (d)). Bar = 100 *μ*m.

The results of histopathological examinations of the kidney at 18 weeks of age are shown in Table 1 and Figure 3. The following findings in the glomerulus, tubule, and interstitium were observed in both the sham group and 1/2 Nx group. Glomerulosclerosis was characterized by an increase in size of the glomerulus and diffuse thickening of the glomerulocapillary wall and the mesangial expansion, showing partly segmental fibrosis in severe cases. Tubular lesions included tubular regeneration, dilatation, and hyaline casts, and interstitial lesions included fibrosis and inflammatory cell infiltration. The histological features of the kidney were not different between the 1/2 Nx group and the sham group; however, a more progressive pathological degree was observed in the 1/2 Nx group.

## 4. Discussion

Glomerular lesions occurring due to renal mass reduction have been demonstrated [15, 16]. For example, kidney damage is exacerbated by nephrectomy in streptozotocin-diabetic rats [17] and Zucker fatty rats [18]. Therefore, unilateral nephrectomy is an effective method to accelerate the manifestation of renal alterations. In the present study, we evaluated the effects on renal function of SDT fatty rats subjected to unilateral nephrectomy, as well as the renal morphology of these animals.

Proteinuria, increased blood urea, hypertension, and glomerular sclerosis, which are considered main characteristics of CKD, were observed in the 1/2 Nx group at a younger age compared with the sham group.

Compensatory hypertrophy of the remnant kidney after unilateral nephrectomy is well recognized [19]. This phenomenon is accompanied by pathological changes that lead to reduced renal function, although the weight of kidneys has not been used as an indicator of renal dysfunction [19]. In agreement with these findings, the remnant kidney in the 1/2 Nx group was significantly heavier than those in the sham group, and histopathological examinations of remnant kidneys in the 1/2 Nx group showed degenerative changes such as glomerulosclerosis in the glomerulus and interstitial inflammation in the interstitium. These histopathological findings were not observed in normal Sprague Dawley rats.

Hyperglycemia is a known stimulus for renal hypertrophy and its association with reduction in renal mass affects this hypertrophy [15, 16, 20]. In the present study, levels of plasma glucose were similar among groups. This result suggests that the contribution of hyperglycemia to the exacerbation of renal function in the 1/2 Nx group is likely low.

Altered lipid metabolism influences the development and progression of glomerular injury [21, 22]. Lipid abnormalities in the 1/2 Nx group, which accompany a reduction in renal mass, may contribute to progressive glomerular damage.

Hypertension is a hemodynamic characteristic of CKD, which could accelerate the progression of renal dysfunction by worsening glomerular injury and proteinuria [23]. It is possible that the increased blood pressure observed in the 1/2 Nx group in this study contributed to the accelerated glomerular injury and consequently led to marked proteinuria. Proteinuria has been considered a strong predictor of kidney disease outcome [24]. In addition, urinary biomarkers such as kidney injury molecule-1 (KIM-1) and N-acetyl-*β*-D-glucosaminidase (NAG) have been reported [25, 26]. In the further study, measuring these biomarkers may be useful to assess the severity of diabetic kidney damage.

In conclusion, induction of 1/2 Nx in SDT fatty rats led to functional and morphological damage of the remnant kidney and to hypertension, which are considered main characteristics of chronic kidney disease, at a younger age. The early onset of diabetic nephropathy in SDT fatty rats is an advantage for CKD research. The SDT fatty rat has promise in the further elucidation of the pathogenesis of human diabetic nephropathy and in new drug discovery.

## Figures and Tables

**Table 1:** Histopathological findings of kidney in sham group and 1/2 Nx group.

Findings		Group													
		1/2 Nx									Sham				
	Animal number	1	2	3	4	5	6	7	8		9	10	11	12	13
Glomerulus															
Glomerulosclerosis		+	+	2+	+	+	2+	+	+		±	±	±	±	−
Tubule															
Regeneration		+	2+	2+	2+	2+	2+	2+	2+		+	+	+	+	+
Dilatation		2+	2+	+	2+	+	2+	2+	2+		+	+	±	+	+
Hyaline cast		+	+	±	±	+	±	+	+		+	+	±	+	±
Deposit, hyaline droplet		±	−	−	−	−	−	−	−		−	−	−	−	−
Armanni-Ebstein change		+	+	+	+	+	±	±	−		+	+	+	+	+
Mineralization		−	+	+	+	±	+	+	+		+	+	+	−	+
Interstitial															
Fibrosis, interstitial		−	+	−	−	−	+	−	+		−	+	±	−	±
Infiltration, inflammatory cell, interstitial		+	+	2+	2+	+	2+	2+	2+		±	−	+	±	+

−: negative; ±: very slight; +: slight; 2+: moderate; 3+: severe.
